# The mitochondrial genome of *Ephestia elutella* (Insecta: *Lepidoptera*: Pyralidae)

**DOI:** 10.1080/23802359.2018.1436993

**Published:** 2018-02-10

**Authors:** Qiongyou Liu, Xiaohong Jiang, Xiaohui Hou, Hong Yang, Wenlong Chen

**Affiliations:** a Department of Basic Medical Sciences, Zunyi Medical University, Zunyi, Guizhou, P. R. China;; b Institute of Entomology, College of Tobacco Science, Guizhou University, Guiyang, Guizhou, P. R. China;; c Guizhou Provincial Key Laboratory for Agricultural Pest Management of Mountainous Region, Guiyang, Guizhou, P R China;; d Special Key Laboratory for Development and Utilization of Insect Resources of Guizhou, Institute of Entomology, Guizhou University, Guiyang, Guizhou, P. R. China

**Keywords:** Mitochondrial genome, Pyralidae, phylogenetic, *Ephestia elutella*

## Abstract

In this study, the complete mitochondrial genome of the tobacco moth *Ephestia elutella* was sequenced and analyzed. The mitochondrial genome is 15345 bp long and contains 13 protein-coding genes, two rRNA genes, 22 tRNA genes, and one control region. Twenty-three genes were found to be encoded by the majority strand and the other 14 genes by minority strand, those is similar to that of other insects. The nucleotide compositing of the majority strand are 38.6% of A, 11.77% of C, 42.05% of T and 7.58% of G. The phylogenetic analysis by Maximum-likelihood (ML) method revealed that the *E. elutella* was close to the same genus insect *Ephestia kuehniella.*

The tobacco moth *Ephestia elutella* (Hübner) (*Lepidoptera*: Pyralidae) is one of the main pests in stored tobacco, and it is cosmopolitan in distribution (Ashworth 1993). However, little information about its genetic characteristic has been reported. Therefore, we determined to sequence the complete mitochondrial genome of *E. elutella* using the *De Novo* sequencing techniques strategy, with the purpose to studying biogeographic, molecular, and population studies. The adult *E. elutella* was obtained from tobacco warehouses in Guiyang Tobacco Redrying Factory, Guiyang, China (GPS 26.52094°N, 106.67497°E), and then was reared on toacco in lab. The voucher specimens are deposited in Institute of Entomology, Guizhou University, Guiyang, China (GZU-LE-000019). The fourth larvae stage of *E. elutella* were washing with 70% ethanol first, and then those were stored in 95% ethanol, the mitochondrial DNA was extracted using *De Novo* sequencing library, and DNA sequencing at TGS (Total Genomics Solution Institute, Shenzhen, China).

The entire sequence of *E. elutella* mitochondrial genome (Genbank accession no. MG748858) is 15345 bp in length, consisting of 13 protein-coding genes (PCGs), 2 ribosomal RNA genes (rRNA), 22 transfer RNA genes (tRNA), and 1 control region. Twenty three genes (*trnM*, *trnI*, *nad2*, *trnW*, *cox1*, *trnL2*, *cox2*, *trnK*, *trnD*, *atp8*, *atp6*, *cox3*, *trnG*, *nad3*, *trnA*, *trnR*, *trnN*, *trnS1*, *trnE*, *trnT*, *nad6*, *cytB*, and *trnS2*) were found to be encoded by the majority strand and the other 14 genes (*trnQ*, *trnC*, *trnY*, *trnF*, *nad5*, *trnH*, *nad4*, *nad4L*, *trnP*, *nad1*, *trnL1*, *rrnL*, *trnV*, *and rrnS*) by minority strand, those is similar to that of other insects (Lin et al. 2017; Living Prairie Mitogenomics Consortium 2017; Singh et al. 2017). Overall nucleotide compositions of the majority strand are 38.6% of A, 11.77% of C, 42.05% of T and 7.58% of G, with an AT content of 80.65%.

All the protein-coding genes begin with ATN start codon except the *cox1*,which starts with CGA similar to other *Lepidoptera* insects (Singh et al. 2017); *cox3*, *nad4*, *nad4L*, and *cyt b* genes employing ATG; while the rest using ATA as a start codon. Three PCGs (*cox2, nad5, and nad4*) have aberrant single-nucleotide (T) stop codons, and the other PCGs showed canonical stop codon pattern TAA. The *lrRNA* is located between *tRNAL1* and *tRNAV*, whereas *srRNA* is accommodated between *tRNAV* and control region. The 22 tRNA genes vary from 63 to 70 bp in length. The secondary structure of tRNAs exhibited typical clover-leaf structure similar to other insect species. The size of the control region is 319 bp (44.2% A, 1.25% G, 49.84% T, 4.7% C, with an AT content of 94.04%), and it located between *srRNA* and *tRNAM*, and the gene order around the control region is tRNAV-srRNA-control region-tRNAM-tRNAI-tRNAQ, and the order model was similar to other *lepidopteran* insects (Singh et al. 2017), and it is different to Coleopteran insects (Zhang and Hewitt 1997).

The phylogenetic tree of *E. elutella* was constructed with the complete mtDNA sequences from 13 pyralidea moth by MEGA 7.0 (Kumar et al. 2016) using maximum-likelihood (ML) methods(Chen et al. 2016; Liu et al. 2018). As shown in Figure 1, the *E. elutella* was close to *Ephestia kuehniella.* Thus, this result supported the monophyly of *E. elutella.*

**Figure 1.**
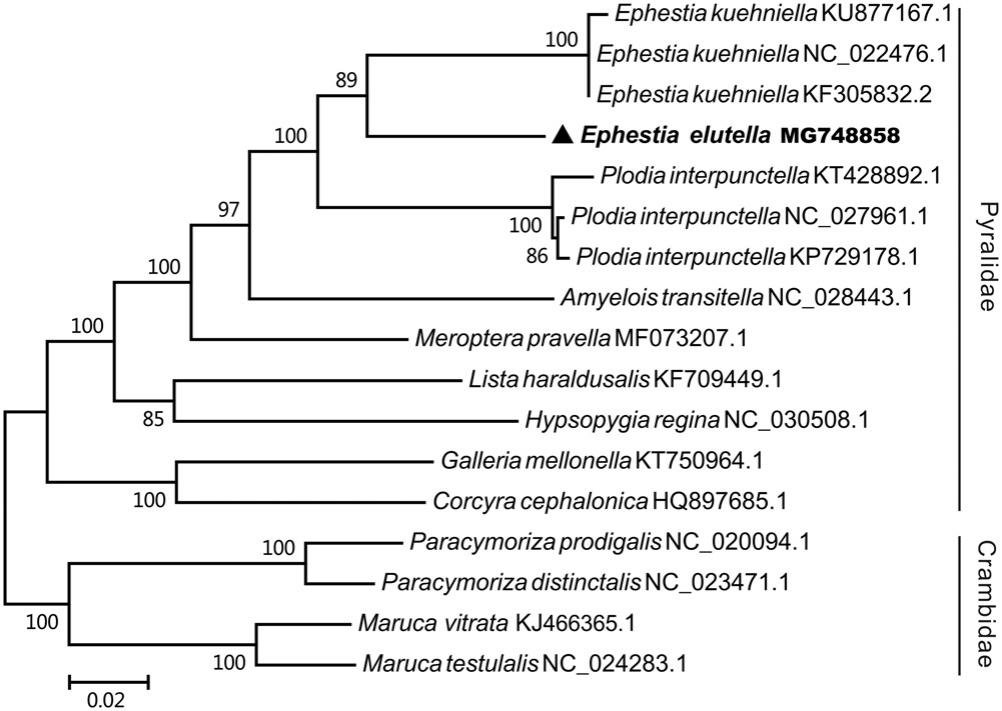
The maximum-likelihood phylogenetic tree of 13 pyralidea species. The nucleotide sequences of the complete mitochondrial genome were downed from GenBank. The phylogenetic tree was constructed by MEGA 7.0 and Bootstrap support is shown at nodes.

## References

[CIT0001] Ashworth JR. 1993 The biology of *Ephestia elutella*. J Stored Prod Res. :199–205.

[CIT0002] Chen H, Deng S, Yang H, Ma X, Zhu C, Huang H, Li G. 2016 Characterization of the complete mitochondrial genome of *Priacanthus tayenus* (Perciformes: *Priacanthidae*) with phylogenetic consideration. Mitochondrial DNA Part B. 1:243–244. 10.1080/23802359.2016.1156493 PMC7871866 33644351

[CIT0003] Kumar S, Stecher G, Tamura K. 2016 MEGA7: molecular evolutionary genetics analysis version 7.0 for bigger datasets. Mol Biol Evol. 33:1870–1874. 27004904 10.1093/molbev/msw054 PMC8210823

[CIT0004] Lin ZQ, Song F, Li T, Wu YY, Wan X. 2017 New mitogenomes of two Chinese stag beetles (Coleoptera, *Lucanidae*) and their implications for systematics. J Insect Sci (Online). 17:63. 10.1093/jisesa/iex041 PMC5469381 28931158

[CIT0005] Liu Q, Jiang X, Hou X, Cai R, Tan J, Chen W. 2018 The mitochondrial genome of *Lasioderma serricorne* (Coleoptera, *Anobiidae*). Mitochondrial DNA Part B. 3:64–65. 10.1080/23802359.2017.1422400 PMC7799969 33474067

[CIT0006] Living Prairie Mitogenomics Consortium 2017 The complete mitochondrial genome of the lesser aspen webworm moth *Meroptera pravella* (Insecta: *Lepidoptera*: Pyralidae). Mitochondrial DNA Part B. 2:344–346. 10.1080/23802359.2017.1334525 PMC7800001 33473822

[CIT0007] Singh D, Kabiraj D, Sharma P, Chetia H, Mosahari PV, Neog K, Bora U. 2017 The mitochondrial genome of Muga silkworm (*Antheraea* assamensis) and its comparative analysis with other *lepidopteran* insects. PLoS One. 12:e0188077. 29141006 10.1371/journal.pone.0188077 PMC5687760

[CIT0008] Zhang DX, Hewitt GM. 1997 Insect mitochondrial control region: a review of its structure, evolution and usefulness in evolutionary studies. Biochem Systemat Ecol. 25:99–120.

